# A Scoping Review of Peer Navigation Programs for People Living with HIV: Form, Function and Effects

**DOI:** 10.1007/s10461-022-03729-y

**Published:** 2022-06-07

**Authors:** Timothy Krulic, Graham Brown, Adam Bourne

**Affiliations:** 1 Australian Research Centre in Sex, Health and Society, La Trobe University, Melbourne, Australia; 2 Living Positive Victoria, Melbourne, Australia; 3 The Centre for Social Impact, UNSW, Sydney, Australia; 4 Australian Research Centre in Sex, Health and Society, La Trobe University, Building NR6, Bundoora, VIC 3086 Australia

**Keywords:** HIV, Peer Navigation, Patient Navigation, Peer support, Quality of life, Adherence, Scoping review.

## Abstract

This scoping review maps recent research into peer navigation programs for people living with HIV. Four databases were systematically searched in June 2020. Results were screened according to defined criteria and were not restricted to any design, outcome or country. Six papers drew from randomised control trials, five from quasi-experimental or pragmatic trials, and four panel, eight qualitative, three mixed method and one cross-sectional designs were included for review. Programs incorporated health systems navigation and social support. Authors provided strong theoretical bases for peers to enhance program effects. Studies primarily reported program effects on continuum of care outcomes. Further research is required to capture the role HIV peer navigators play in preventing disease and promoting quality of life, mental health, and disease self-management in diverse settings and populations. Peer programs are complex, social interventions. Future work should evaluate detailed information about peer navigators, their activities, the quality of peer engagement as well as employee and community support structures to improve quality and impact.

**Supplementary information:**

The online version contains supplementary material available at 10.1007/s10461-022-03729-y.

## Background

The involvement of people living with HIV in their own care and wellbeing is a hallmark of responses to HIV and AIDS in many places around in the world. The meaningful involvement of key populations of people living with and affected by HIV, including gay men and their allies, sex workers and people who inject drugs has underpinned successful national HIV strategies, with peer and community-based responses now recognised globally as critical to meeting contemporary efforts to end the epidemic [1, 2].

For over 40 years, researchers have described and assessed the effectiveness of peer and community-based responses in a number of areas of health [3–5]. While diverse in its traditions and conceptualisations, the role of peers in health promotion relies on the affinity, connection and experiences peers share with their communities to enable effective communication, education, advocacy and social support [4, 6]. Since the arrival of antiretroviral therapy (ART) most research in HIV has focused on people living with HIV assisting peers in healthcare settings to live well with chronic manageable illness [3, 7]. The engagement of communities as partners, leaders and decision-makers in national health systems and HIV strategies, however, remains limited. A lack of contemporary investment in these responses is a major barrier to meeting global elimination targets for HIV [8].

People living with HIV have also taken up the role of health systems navigation. As a patient-centred model of care developed in cancer treatment and other areas of healthcare, peer navigators provide guidance and support through complex health systems, acting as a bridge between clinical and community services and social supports [9–12]. As peers are resourced with training, supervision, pay and other employment conditions, a growing body of practice-based evidence, guidelines and standards now position peer navigation as a more formal occupation for peers working alongside other healthcare practitioners in the HIV care and support sector, particularly in high-income settings [13–18].

Evidence reviews which have systematically assessed the impact of HIV peer navigation programs and similar peer interventions have largely focused on the efficacy of programs to strengthen the treatment cascade [3, 7, 10, 19, 20]. The dual benefits of disease and primary prevention that highly effective ART affords has driven the establishment of the ’90 90 90’ global treatment targets [21]. By 2020, according to global estimates, 81 per cent of people living with HIV knew of their positive status, 67 per cent of people diagnosed were receiving sustained treatment and 59 per cent of those on treatment were virologically suppressed [22]. Although high quality designs have shown for some time that peer navigation and peer interventions can improve these continuum of care outcomes for individuals in experimental settings, until recently results have lacked consistency [7].

Diverse results from randomised control trials (RCTs) evaluating peer navigation programs are challenging to interpret. This is in part because many published reports do not provide full descriptions of how programs operate to achieve desired effects [6, 10]. The most recent systematic review and metanalysis, which limited results to studies evaluating in-person and individually tailored peer navigation and support interventions, found more consistent evidence that this approach was superior to standard care in supporting HIV continuum outcomes [7]. The influence of other program mechanisms and qualities such as the nature of peer navigator activities and their characteristics, skills, training and employment and support structures are less well understood [6, 10]. Recent systematic reviews also have not considered evidence from qualitative and non-experimental designs that may help address factors relevant to the implementation and improvement of HIV peer navigation programs.

Improving quality of life and chronic healthcare outcomes for people living with HIV is now a major focus of the global HIV response [23]. Addressing stigma, social isolation, insecurity, non-communicable diseases like depression and anxiety as well as the detection and treatment of opportunistic infections, particularly in resource-poor settings, will be vital to improve not only the quality of life that people living with HIV experience but also global treatment goals and targets for the reduction of new infections and deaths [8]. Despite this, fewer evidence reviews have considered results from studies which show how peer navigation programs can improve health and wellbeing outcomes beyond the treatment cascade [3, 7].

This scoping review examined the extent and nature of research into peer navigation services for people living with HIV. It aimed to [1] define how HIV peer navigation programs are conceptualised and operationalised within the research literature; [2] determine what health outcomes programs aim to effect and how these are framed, captured and reported; and [3] identify priorities for research and improving the quality and impact of services. This detailed synthesis of form, function and reported outcomes will be useful to program planners, policy makers and researchers. Identifying the underlying mechanisms for action and the factors that contributed to program effectiveness in different contexts will help to facilitate improved design, scale-up, impact and evaluation of peer navigation programs in diverse settings and populations of people living with HIV.

## Methods and Design

Our review broadly followed frameworks for scoping reviews outlined by Arksey and O’Malley and refined by Levac et al. [24, 25]. PRISMA guidelines [26] were followed for reporting (see Supplementary Material 1 for a completed checklist).

### Inclusion Criteria

This study included original peer-reviewed research papers which investigated the effects, context, and delivery of HIV peer navigation programs. Evidence reviews and papers reporting protocols and baseline data were not included. Studies were not restricted to any design, outcome or geographic region and targeted people living with HIV 18 years or older.

We screened for papers that reported use of peer navigation or similar services. To support the overall aims of the scoping review we used broad search terms that would identify studies that investigated peer navigation services even if the term was not used to describe the intervention or program. If authors did not use the term peer navigation, for a paper to be included navigators were HIV positive and offered in-person, tailored support to individuals within a formalised role (rather than personal relationships and social networks) and provided links to social support, healthcare or community services.

### Identifying Relevant Studies

We searched articles indexed in Embase, MEDLINE, Psych Info and CINAHL on June 22, 2020. Results were limited to papers published in English. Our search was restricted from 2015 onwards, when international targets were first established to treat 90 per cent of people living with HIV [21]. This captured HIV peer navigation programs and studies operating in the contemporary ‘treatment cascade’ environment.

Our search strategy developed keywords and terms relevant to people living with HIV and peer navigation. Keywords and search terms included: people, men or women living with HIV or HIV/AIDS; HIV or HIV/AIDS patients, support or care; PLHIV, PLHWA, Acquired immunodeficiency syndrome and HIV seropositivity. The above terms were combined with peer navigation, counselling, support, help, mentor or education and patient navigation or advocacy. See Supplementary Material 2 for a sample search strategy.

Two reviewers independently screened subsets of titles and abstracts using the inclusion criteria to identify potentially relevant studies. The study team then met several times to discuss discrepancies and review the search strategy and inclusion criteria based on results. Following initial screenings of abstracts and titles, the full papers of articles were reviewed for relevance against finalised criteria. Additional papers or sub-studies published using data from the studies captured by our search were included for analysis if they met inclusion criteria. The authors of papers were not contacted for clarification or additional information as our review aims were concerned with assessing how information about peer navigation programs and their outcomes were framed, captured and reported in published materials.

### Data Extraction and Analysis

All members of the study team collaboratively drafted a data charting form containing 10 variables relating to our research aims. We aimed to capture data on research settings, methods, aims, target populations and reported outcomes as well as descriptions of program personnel, peer navigator roles, program activities and logics or underlying theorical frameworks used to explain program effects.

The first author imported the full text of papers into NIVO software, generating first codes and then summaries related to each of these variables. Codes and the data charting form were both reviewed and modified in meetings between the study team as this analysis was completed. Summaries were then further refined during meetings with attention paid to commonalities and connections across the sample and implications within the broader research literature to determine research priorities and recommendations for program implementation and improvement.

Our review appraised factors related to the conceptualisation and implementation of peer navigation interventions to provide recommendations for the improvement of program quality and impact. No formal critical appraisal, assessment of the risk of bias or statistical metanalysis of quantitative data were proposed due to the heterogeneity of study designs.

## Results

Twenty-seven papers investigating nineteen unique peer navigation programs and implementation settings were included in this review [27–53]. Our database search identified 1143 records. Following the removal of duplicates, the abstracts and titles of 668 papers were screened, followed by the full text and supplementary materials of 127 papers, resulting in 644 exclusions. Three papers that published data related to studies identified by our search met criteria and were included for review. Table 1 provides a summary of the publication details, design, participants and outcomes for each study included in the review.

**Table 1:** Characteristics of included studies

Author, date, country (ref) design.	Implementation setting	Target population	Outcomes
Adams J, 2020, USA (27). Pragmatic trial.	Urban clinic.	PLHIV, clinic patients (n = 954).	L&R
Cabral H, 2018, USA (28). RCT.	Urban clinic.	PLHIV from ethnic minorities (n = 348).	L&R; VS; Self-efficacy; HIV knowledge; HRQoL
Cataldo F, 2017, Malawi (29). Qualitative.	Urban and rural clinics and community health organisations.	Pregnant and breastfeeding women living with HIV (n = 24).	ART
Chang LW, 2015, Uganda (30). Randomised pragmatic trial.	Rural clinic.	Treatment naïve PLHIV (n = 442).	ART; L&R; HIV prevention.
Chevrier C, 2016, India (31). Qualitative.	Urban community health organisation.	PLHIV sex workers (n = 22) interventionists (n = 6) community organisation representatives (n = 12).	Health service engagement; community and social support engagement
Cunningham W, 2018, USA (32). RCT.	Urban, incarcerated and post release.	Men and trans women living with HIV being released from jail (n = 356).	VS; ART adherence; L&R; treatment and adherence knowledge; drug use; health service utilisation.
Giordano TP, 2016, USA (33). RCT.	Urban hospital	PLHIV hospital outpatients, out of care (n = 460)	VS; L&R; HRQoL; health service utilisation.
Graham SM, 2015, Kenya (34). Qualitative.	Rural clinic	MSM living with HIV (n = 30) and healthcare providers (n = 29).	ART
Griffith D, 2019, USA (35). Pragmatic trial.	Urban clinic	Youth and young PLHIV aged 18–30, history of mental health diagnosis, substance use or adherence issues (n = 137)	VS; L&R
Hosseinipour M, 2017 Malawi (36); Phiri S, 2017, Malawi (45). RCT.	Urban and rural clinic and community health organisation.	Pregnant and breastfeeding women living with HIV (n = 1272).	VS; L&R
Karwa R, 2017, Kenya (37). Mixed methods.	Urban hospital	PLHIV in hospital care (n = 1,357).	L&R; ART
Koneru A, 2017, Tanzania (38). Cross-sectional.	Urban clinic	Women living with HIV (n = 399).	Cancer prevention
Lifson AR, 2017. Ethiopia (39). Panel/longitudinal.	Rural clinic	PLHIV newly enrolled in clinical care (n = 142).	L&R; HRQoL; HIV knowledge
Maulsby C, 2015, USA (40). Panel/longitudinal	Urban and rural community health organisations	PLHIV, MSM, newly diagnosed, incarcerated, out of care or on a Medicaid plan (n = 2,615).	L&R; VS
Monroe A, 2017, Uganda (41). Qualitative.	Rural clinics	Treatment naïve PLHIV, peer support workers and clinic staff (n = 75)	ART; L&R; HIV prevention
Minick SG, 2018, USA (42). Qualitative.	Urban hospital	PLHIV hospital outpatients, out of care (n = 25) and interventionists (n = 9).	VS; L&R
Myers JJ, 2018, USA (43). RCT.	Urban incarcerated and post-release setting	PLHIV exiting jail with current or former experience of drug use (n = 270).	VS; L&R; HIV prevention; AOD risk behaviour; health service utilisation
Odiachi A, 2020, Nigeria (44). Mixed methods.	Rural clinics	Mothers and pregnant women living with HIV (n = 100) and expert mothers (n = 37).	VS; L&R; HIV prevention.
Pitpitan EV, 2020, Mexico (46). Qualitative.	Urban community health organisation	PLHIV; people who use drugs, sex workers, MSM, trans women. Service providers (n = 8)	L&R
Ryerson Espino SL, USA (47). Qualitative.	Urban and rural clinic and community health organisations	Women living with HIV from ethnic minorities. Program administrators, intervention staff, evaluators and program partners from (n = 11) implementing organisations.	L&R
Reback CJ, 2019, USA (48); Reback CJ, USA, 2019 (49). Panel/longitudinal.	Urban community health organisation	Trans women from ethnic minorities living with HIV (n = 129)	VS; L&R; ART.
Sam-Agudu NA, 2017, Nigeria (49); Sam-Agudu NA, 2017, Nigeria (51). Non-randomised control trial.	Rural clinic	Mothers and pregnant women living with HIV (n = 497).	HIV prevention; VS; L&R
Sam-Agudu NA, 2018, Nigeria (52). Qualitative.	Rural clinics	Mothers and pregnant women living with HIV. Expert mothers (n = 36).	VS; L&R; HIV prevention.
Steward WT, 2018, South Africa (53). Mixed methods.	Rural clinic	PLHIV, newly diagnosed clinic patients (n = 35), navigators (n = 4) and clinic providers (n = 5).	L&R; ART; HIV prevention.
**Legend** n = number. MSM = men who have sex with men. PLHIV = people living with HIV. L&R = linkage and retention in care. VS = virological suppression. HRQoL = health-related quality of life. ART = ART initiation and adherence.			

### Research Design, Settings and Participants

Our search captured a range of study designs, including six papers that reported results from four RCTs, five papers drawing from four quasi-experimental or pragmatic trials, and four panel, eight qualitative, three mixed method and one cross-sectional designs. As indicated in Table 1, this includes related papers and sub-studies reporting data from trials investigating the same peer navigation program or implementation setting.

As shown in Table 2, nine studies were based in the United States. Our search also found research investigating peer navigation programs and implementation settings in Malawi, Uganda, Kenya, Tanzania, Ethiopia, Nigeria, South Africa, India and Mexico. All five of the studies conducted entirely in rural areas were in sub-Saharan Africa. Three studies conducted in the United States and Malawi collated data from multiple sites across rural and urban areas. Ten programs were delivered by clinics or healthcare providers, three by community health organisations and two studies collated and compared data from programs operating across both clinical and community settings (see Table 2). Two programs operated in incarcerated or post-release settings.

**Table 2:** Summary of study characteristics

		n
Country of investigation	USA	9
	Malawi	1
	Nigeria	1
	Uganda	1
	Ethiopia	1
	Kenya	2
	South Africa	1
	India	1
	Tanzania	1
	Mexico	1
	Total	19 ^a^
Location	Urban	11
	Rural	5
	Urban and rural	3
	Total	19 ^a^
Implementing organisation(s)	Community health organisation	3
	Clinic	8
	Hospital	2
	Clinic and community health organisation	2
	Correctional or post-release services	2
	Total	17 ^a b^
Study designs	RCT	6
	Quasi-experimental or pragmatic trial	5
	Longitudinal/panel	4
	Mixed Methods	3
	Cross-sectional	1
	Qualitative	8
	Total	27
Term or title for peer intervention	Peer navigation	11
	Patient navigation	1
	Peer support	1
	Mentor or expert mothers	2
	Peer Mentor	1
	Multiple	1
	Local language term	2
	Total	19 ^a^
^a^ total combines related studies investigating the same program or implementation setting. ^b^ only includes implemented interventions and programs.		

Table 1 shows that our review found studies investigating programs proposed for or targeting key populations of people living with HIV such as women and mothers, men who have sex with men, trans women, sex workers, people who use drugs, people being released from incarceration, racial and ethnic minorities and youth and young people. Papers also targeted people living with HIV identified as having additional risk-factors such recent diagnosis, loss to care or substance use (see Table 1). Participants and key informants included peer workers, service providers and clinicians.

### HIV Peer Navigation Concept and Operation

Our search identified a range of titles and terms to describe peer navigation roles and programs. Peer navigation was the most common term, however, programs and roles very similar in scope and function were also called patient or health navigation, peer or patient mentoring, peer support, community health workers, ‘mentor’ or ‘expert’ mothers as well as terms drawing on local language and traditions for peer and community-based support for health and wellbeing (see Table 2).

#### A new iteration of peer support and patient navigation

Peer navigation was closely linked to the concept of patient or health systems navigation. As a model of care well-established in healthcare systems and the broader research literature, it was common for authors [32, 33, 37, 38, 42, 43, 46, 48, 49] to draw on the principles and approaches underpinning patient navigation when describing the overarching aims and functions of peer navigation programs. Namely, that peer and patient navigation provide linkages and patient-centred support to overcome health system barriers, improving healthcare engagement and clinical care outcomes. In this way, peer navigation was often framed as a type or recent adaptation of patient navigation. Researchers that used this framing positioned peers as logical interventionist to take up these roles, noting that patient navigation is generally performed by lay workers and paraprofessionals, and that peers possess qualities and experiences that may enhance their effectiveness [32, 33, 42, 46, 48, 49]. Studies which did not explicitly conceptualise programs as peer or patient navigation [29, 36, 38, 39, 44, 45, 50–52] alternatively built on traditions of peer and lay health worker participation in health promotion for people living with HIV. These authors generally cited evidence that peer education and support are widespread in healthcare and that these interventions are effective for HIV prevention and improving clinical care outcomes for people living with HIV.

To inform the design and implementation of peer navigation programs our review further identified how authors defined peers and their activities, as well as theories that were tested or developed which would explain the mechanisms through which peer navigators effected health outcomes. For ease of reporting, program qualities for related papers and sub-studies are reported together across Tables 3 and 4 to provide a total of nineteen peer navigation programs and settings for implementation.

**Table 3:** Peer navigator characteristics, conditions and development

Study	Peer characteristics	Pay and conditions	Training and development	Management and supervision
Adams, Judith. 2020 (27).	PLHIV	Employed. Pay and hours not reported.	Brief description of on-job training.	Not Reported.
Cabral H, 2018 (28).	PLHIV; racial or ethnic background; clinic patient.	Employed. Hours or pay not reported.	Brief on-job training clarifying expected role and duties. Monthly calls providing additional training.	Clinical supervision in individual and/or group settings monthly in addition to monthly calls for support with client work.
Cataldo F, 2017 (29); Hosseinipour M, 2017 (36); Phiri S, 2017 (45).	Mothers; PLHIV; clinic patients.	Monthly stipend of US$65 for part-time work.	2-week on-job training on patient education and psychosocial topics.	Twice-yearly refresher trainings and monthly supervision to support client work.
Chang LW, 2015 (30); Monroe A, 2017 (41).	Clinic patients; PLHIV.	Paid a monthly stipend of US$10 plus transport expenses for part-time work.	Detailed description of two-day residential training on role-relevant skills and knowledge.	Supervision from study coordinators who regularly conducted field-based skills reinforcement trainings.
Chevrier C, 2016 (31).	Sex workers; PLHIV.	Volunteer. Services are organised with no regular source of funding.	Not reported.	Not reported.
Cunningham W, 2018 (32).	PLHIV; ethnic or racial background; gender; drug use Incarceration	Employed on full-time salaries with benefits. Pay not reported.	Completed training prior to field work, using a detailed manual of operations.	Daily monitoring, weekly supervision, and periodic auditing of records.
Giordano TP, 2016 (33). Minick SG, 2018 (42).	PLHIV; clinic patients	Volunteer.	Experience in previous mentor position and training on intervention delivery at 2-day workshop.	Periodic fidelity assessments and checklists to guide content delivery.
Graham SM, 2015 (34).	PLHIV; MSM.	Volunteer. Stipends, meals and transport reimbursement were provided.	2 years treatment experience and able to communicate in English or Kiswahili. Brief description of training on role-specific skills and knowledge, conducted over 2 days.	Didactic material and interactive exercises guided delivery. No other information reported.
Griffith D, 2019 (35).	Young adult.	Not reported.	Not reported.	Navigators were part of an interdisciplinary care team. No other information reported.
Karwa R, 2017 (37).	PLHIV; hospital patient.	Employed. Pay and hours not reported.	Participation in a previous study, in which they received training and a year of treatment and adherence counselling experience. Description of 1 week additional and ongoing training on role-specific skills and knowledge provided.	Navigators formed part of an interdisciplinary care team. No other information reported.
Koneru A, 2017 (38).	PLHIV; cervical cancer survivor.	N/A	N/A	N/A
Lifson AR, 2017 (39).	PLHIV.	Monthly stipend of 700 Ethiopian birr (US$37). Hours not reported.	Brief description of initial and refresher training role-relevant skills and knowledge.	Individual supervision from a project coordinator. The community health support workers s met as a group monthly to discuss problems, potential strategies, and lessons learned from client work.
Maulsby C, 2015 (40).	Not reported.	Not reported.	Not reported.	Not reported.
Myers JJ, 2018 (43).	PLHIV; drug use; incarceration.	Employed at part time/casual hours (10–12) across 2 days per week. Pay not reported.	Brief description of trainings provided for role-relevant skills and knowledge.	Ongoing support and clinical supervision.
Odiachi A, 2020 (44). Sam-Agudu NA, 2017 (50). Sam-Agudu NA, 2017 (51). Sam-Agudu NA, 2018 (52).	Mothers; PLHIV.	Monthly stipend of US$50 with flexible working hours.	Required to read and write basic English. Detailed description of training provided on scope of duties and role-relevant skills and knowledge.	Ongoing supervision reinforcing training was provided.
Pitpitan E V, 2020 (46).	PLHIV (non-exclusive); drug use; sex work; MSM; trans experience; gender	N/A	N/A	N/A
Ryerson Espino SL (47)	PLHIV; gender; ethnic or racial background.	A mixture of part-time, stipend and full-time positions across sites. Advice about pay and conditions reported in findings.	An extensive description of training available to peer staff across sites is provided, including role-specific knowledge and skills, general health, social service and employment training.	A detailed description is provided of strategies across sites including weekly individual supervision, refresher training, emotional support and debriefing for client work, cross-support from peers, and peer leadership.
Reback CJ, 2019 (48). Reback CJ, 2019 (49).	PLHIV; trans experience; gender; ethnic or racial background	Not reported.	Detailed description of initial and ongoing training provided on role-relevant skills and knowledge.	Semi-monthly clinical supervision and annual refresher training on skills for client work and monitoring of medical care and records.
Steward WT, 2018 (53).	PLHIV; gender	A small stipend. Workload was managed by limiting 10 clients to each navigator.	Brief description of one-week training on role-specific skills and knowledge. Ongoing training augmented skills or addressed observed deficiencies.	Weekly supervision and biweekly meetings with study team to monitor fidelity and support client work.
**Legend** PLHIV = people living with HIV MSM = men who have sex with men.				

**Table 4:** Program activities and mechanisms

Study	Program name	Activities	Mode of contact	Program theory or hypothesis
Adams, Judith. 2020 (27).	Peer Navigation	Clinical liaison and communication; practical support and material aid.	One home visit for missed appointment.	No underlying theoretical framework or explanation of peer influence reported.
Cabral H, 2018 (28).	Peer Navigation	Linkage and referral; practical support and material aid; information and education; emotional and social support.	7 (60 min every 1–3 weeks) sessions and weekly check-ins by phone or in person which ranged from 30 to 60 min or every 2 weeks for up to 4 months.	Based on social support framework, peers enhance the effect of program activities through greater credibility, trust, empathy and understanding.
Cataldo F, 2017 (29). Hosseinipour M, 2017 (36). Phiri S, 2017 (45).	Mentor or expert mother	Information and education; emotional and social support.	Support available in clinic or during home visits during 2-year trial period. Appointment reminders via phone or home visits.	Peers have more time to support patients with issues of disclosure, stigma and treatment initiation. No underlying theoretical framework or explanation of peer influence reported.
Chang LW, 2015 (30). Monroe A, 2017 (41).	Peer Support	Clinical liaison and communication; linkage and referral; information and education; emotional and social support.	Monthly visits for 12 months. Visits typically occurred in the home but could be arranged at other locations or in clinic.	IMB model used to explain how peers were able to enhance the effect of program activities, motivation and the uptake of information and behavioural skills through greater empathy and understanding.
Chevrier C, 2016 (31).	Ashraya (local language term) volunteers	Coaching and skills building; practical support and material aid; emotional and social support; advocacy.	Accompaniment to healthcare appointments and intervention in instances of discrimination in community. Care facilities were also available at CHO. Duration not reported.	Based on social empowerment theory, social support and advocacy acted as a buffer to negative effects of stigma and discrimination. Qualitative findings established that peers had a higher degree of credibility and that empathy and understanding enhanced the effect of activities.
Cunningham W, 2018 (32).	Peer Navigation	Clinical liaison and communication; practical support and material aid; information and education; coaching and skills building.	1–2-hour sessions were conducted, once during pre-release and in community settings post-release for a 24-week period, including accompaniment to 2 medical care appointments.	Based on social learning theory, peers were trusted sources of information and effective role models for behavioural skills and strategies to overcome stressors and barriers to desired health outcomes.
Giordano TP, 2016 (33). Minick SG, 2018 (42).	Peer Mentoring	Linkage and referral; information and education; coaching and skills building; emotional and social support.	2 in person 20–45-minute sessions in hospital, followed by 5 telephone calls after discharge over the next 10 weeks.	IMB model used to explain how peer mentors enhanced motivation and program activities through role modelling, credibility, trust, empathy and understanding.
Graham SM, 2015 (34).	Peer Navigation and Kiswahili language term Washikaji (meaning ‘‘those who bond or stick together’’).	Information and education; emotional and social support.	In person or by telephone at least weekly during the first month of ART, then at least monthly for the remaining follow-up.	Novel framework proposed that access, information, motivation, and proximal cues to action are necessary to engage participants in care and treatment. Peers enhanced all aspects of the model through role modelling and establishing trust and credibility.
Griffith D, 2019 (35).	Peer Navigation	Clinical liaison and communication;	Phone and text conversations. Duration not reported.	An interdisciplinary care team with specific training in youth-focused care, including peer navigator would better meet needs of youth PLHIV. No underlying theoretical framework or explanation of peer influence reported.
Karwa R, 2017 (37).	Peer Navigation	Linkage and referral; information and education; practical support and material aid.	Navigators met with patients on wards. In-patient and out-patient follow up was then provided. Duration not reported.	Peers enhance program activities through role modeling and greater empathy and understanding. No underlying theoretical framework reported.
Koneru A, 2017 (38).	Peer Navigation	Clinical liaison and communication; linkage and referral; information and education; practical support and material aid; emotional and social support.	Clinic-based appointments, accompaniment and phone call or text reminders. Duration not reported.	Peer navigators were likely to have greater credibility and acceptability among women living with HIV due to sharing similar experiences and backgrounds. No underlying theoretical framework reported.
Lifson AR, 2017 (39).	Peer Community Health Support Worker	Clinical liaison and communication; linkage and referral; information and education; coaching and skills building; emotional and social support.	1–4 visits monthly for a year. Additional phone calls for clinical contact and referrals.	Based on social support and social learning theory, role modelling and greater understanding and empathy from peers enhanced program activities.
Maulsby C, 2015 (40).	Peer Health Navigation or Community Health Outreach Worker	Linkage and referral; information and education.	Outreach and in-reach. Duration varied across sites from 3–6 months, 6–9 months and open-ended.	No underlying theoretical framework or explanation of peer influence reported.
Myers JJ, 2018 (43).	Patient navigation	Clinical liaison and communication; practical support and material aid; coaching and skills building; advocacy.	Initial meeting in-jail upon release followed by in-community and accompaniment to medical, court and other appointments. Clients utilized 9 h per month in months 1 and 2 vs. 2.5 h per month from months 6 through 12).	Drawing from social support theory and patient-centered perspectives, peer enhance effects of program activities through role modelling, empathy, credibility and trust.
Odiachi A, 2020 (44). Sam-Agudu NA, 2017 (50). Sam-Agudu NA, 2017 (51). Sam-Agudu NA, 2018 (52).	Mentor or Expert Mother	Clinical liaison and communication; linkage and referral; information and education; coaching and skills building; emotional and social support.	Home visits every 2 weeks after linking with clients at clinic. Visits continued for 12 months after delivery of infants, with additional calls or visits in the event of missed clinic appointments.	Peers enhanced program activities through greater understanding and empathy. No underlying theoretical framework reported.
Pitpitan E V, 2020 (46).	Peer Navigation	Clinical Liaison and communication; coaching and skills building; linkage and referral; emotional and social support.	N/A	Incorporating elements of social support frameworks, peers enhance program activities through greater trust, credibility and empathy
Ryerson Espino SL (47)	Varied across study sites and locations: peer community; health outreach workers; patient navigators; peer educators; peer advocates; peer counsellors; p*romotoras; p*eer specialists; peer client assistants; peer lifeguards	Coaching and skills building; practical support and material aid; emotional and social support; advocacy.	Varied across site and locations. In-person sessions, accompaniment to appointments, outreach and phone contact described. Duration not reported.	Across a range of proposed programs and activities peers would empower and motivate desired health outcomes as role models. No underlying theoretical framework reported.
Reback CJ, 2019 (48). Reback CJ, 2019 (49).	Peer Navigation	Linkage and referral; coaching and skills building; practical support and material aid.	Unlimited in-person contact including accompaniment to appointments. The frequency of contacts titrated down after the first quarter of care.	Based on social learning theory, peers enhanced program activities as role models.
Steward WT, 2018 (53).	Peer navigation	Linkage and referral; coaching and skills building; information and education; clinical liaison and communication.	At least one in-person meeting and one check-in by text or phone for 4 months. Additional contacts were encouraged when needed.	Incorporating elements of social learning theory and social support frameworks, peers enhanced through role modelling and developing a high level of trust, credibility and understanding.

#### Peer navigator characteristics and roles

Our review aimed to clarify the definition of a peer navigator by restricting inclusion to programs involving people living with HIV as navigators, except in cases where the term peer navigation was explicitly used by authors.

Researchers generally incorporated information about the key characteristics, circumstances and experiences of peer navigators to establish their peer status or discuss the theoretical basis, operation and effects of programs. There were only two studies that did not provide enough detail in published materials about the selection of peer navigators to determine whether living with HIV was a factor in establishing peer status (see Table 3). One program proposed that the selection of peer navigators would include but not be limited to people living with HIV. Otherwise, sub-group characteristics constituted peer status in addition to living with HIV. These included gender, sexuality, trans experience, ethnic or racial background, history of drug use or incarceration, taking ART, and being a mother, a client at the same clinic or open and willing to discuss living with HIV (see Table 3).

Reported requirements for formal qualifications or levels of education were minimal (see Table 3), with emphasis placed on the skills and experiences navigators were likely to have as peers. Studies which operationalised or evaluated programs consistently included at least some information about the training made available to peer navigators (see Table 3). Most often these were short courses or on-the-job training focused on content in-line with expected duties. As shown in Table 3, eleven studies reported information about supervision and organisational support for peer navigation roles. Supervision most commonly took the form of a program coordinator reinforcing training and providing feedback on performance and client work.

It was possible to determine whether peer navigators were volunteers, employed or paid a stipend for fourteen studies, however, this information was not always available or uniformly reported (see Table 3). Among the authors who indicated that peer navigators were employed, three did not include information about rates of pay or hours worked. The authors of three studies did not include any information about the pay and conditions of peer navigators.

#### Program activities

Our review identified activities that were common for peer navigators to perform as well as the mode, duration and mechanisms of peer engagement.

A wide range of modalities for peer navigator and client contact were envisioned and are summarised in Table 4. These included face-to-face support sessions at the premises of implementing clinics, hospitals, community health organisations and correctional facilities, outreach in community and the homes of clients as well as accompaniment to appointments and voice or text contact via phones, particularly for follow-up appointments or reminders. The duration of client contact with programs was not always reported, but generally, peer navigation interventions were short-term and intensive, involving a high frequency or unlimited amount of contact across several modes for less than 6-months. It was less common for demand or program protocols to extend beyond 6-months, however, two programs focusing on reproductive and maternal health outcomes extended beyond one year (see Table 4). Four programs described brief interventions with limited contact including one in which peer navigators communicated with clients exclusively through phone calls or text.

As shown in Table 4, education and the provision of information were the most common duties for peer navigators to undertake, followed by health service linkage and referral. Communication and clinical liaison activities were also consistently described by researchers, which included reminders and follow-up for missing appointments and in some cases providing feedback about client experiences. Eleven studies outlined emotional and social support and ten identified coaching and skills building as part of the work of navigators. Programs in nine studies provided practical support and material aid, such as transport, financial support and accompanying clients to appointments. Only the authors of three studies described the work of navigators as advocating on behalf of clients in healthcare settings or instances of poor treatment.

### Program mechanisms

Authors’ discussions of the theoretical foundation for these activities to support health outcomes incorporated elements of social learning theory and social support frameworks, the information-motivation-behavioural skills (IMB) model for behaviour change as well as patient-centred, strengths-based and social empowerment perspectives (see Table 4). Researchers also offered explanations as to why peers living with HIV may be uniquely skilled at performing these activities or more effective at influencing desired outcomes. Although an underlying framework was not always identified, program theories, hypotheses and mechanism centred on the ability of navigators to build on their shared experiences, circumstances or affinity with peers to establish trust, credibility, empathy and understanding or inspire role modelling, motivation and empowerment.

As outlined in Table 4, it was most common for authors to highlight the higher degree of credibility and trust peers are likely build with people living with HIV. Trust was linked to peers belonging to the same stigmatised group or being viewed as having credible insight into overcoming challenges, due to having managed similar stressors and life circumstances. People living with HIV were therefore thought to be more likely to uptake information, referrals, behaviours and beliefs promoted by peers.

Similarly, authors argued that navigators can use their greater understanding of peers’ life circumstances to offer the most relevant and helpful information, referrals, strategies and practical support. Peer navigators’ understanding of the lived experience of HIV was also theorised to contribute to their ability to generate empathy with clients and in turn enhance how the emotional and social support they provided was received. As a strong source of emotional support, authors argued that the peer relationship would empower resilience, improve motivation or act as a buffer from the negative effects of stigma and discrimination on mental wellbeing, medical adherence and healthcare engagement. The authors of one paper also suggested that as peers are more resilient and attuned to the nuances of HIV stigma and discrimination that they would be more effective advocates in instances of poor treatment (see Table 4).

Role modelling was another common mechanism of peer navigation programs, identified by the authors of eight studies (see Table 4). Researchers positioned peer navigators who come from similar backgrounds and experiences as clients and patients as having a strong influence on showing them how to overcome their own challenges and stressors. Modelling success and healthy living was also seen as one way in which the peer relationship motivates and empowers people living with HIV to achieve desired health outcomes.

Most often hypotheses and rationales for peer involvement were outlined in descriptions of interventions or discussions of program effects, however, studies with significant qualitative components [31, 34, 41, 42, 53] provided theory development or rich description of these activities and processes. Researchers reporting on four programs [27, 29, 35, 36, 40, 45] did not include a clear justification for peer engagement grounded in a consideration of the influence of peer skills and characteristics on program operation or effects.

### Health Outcomes

Our review found that recent research into peer navigation has predominately focused on programs aiming to strengthen the HIV treatment cascade.

#### Primary health outcomes

The primary endpoints reported by twenty-five papers were continuum of care outcomes (see Table 5). Only two studies, which investigated the acceptability of peer navigator referral to screening for cervical cancer and peer-based assistance for accessing healthcare, community and social support did not investigate a program primarily aiming to influence treatment cascade outcomes.

**Table 5:** Primary health outcomes

Outcome area	Author, date, country (ref). design.	Reported outcomes
Linkage and retention in care	Adams J, 2020, USA (27). Pragmatic trial.	Decrease of no-show appointments over trial period. Peer navigator completed 10 home visits for missed appointments, resulting in 3 same or next-day appointments. Increased communication between members of the care team about the nuances of individual patient behaviour related to keeping appointments also reported.
	Cabral H, 2018, USA (28). RCT.	There was no evidence of difference between intervention and standard of care groups. There was a statistically significant improvement in retention in care for patients who were stably housed at baseline and those who completed all educational sessions in the intervention model. There was also a significant protective effect of increased face-to-face encounters by peers.
	Chang LW, 2015, Uganda (30). Randomised pragmatic trial.	Participants in the intervention arm who were care naïve at baseline were more likely to report being in care and enroll in care during follow up.
	Giordano TP, 2016, USA (31). RCT.	The peer mentoring intervention was no more successful in improving reengagement or retention in care than the control.
	Griffith D, 2019, USA (35). Pragmatic trial.	Higher risk youth living with HIV receiving intervention had better retention than standard of care in an adult clinic. Bi-directional communication with the peer navigator in the program, via either telephone or electronic message, decreased the risk of missed visits.
	Karwa R, 2017, Kenya (37). Mixed methods.	Patient enrolment was lower than estimates of HIV positive patients on ward, which may be due to implementation challenges. Unwanted disclosure was an issue for this program operating on a public ward, which peer navigators were reported to be skilled at overcoming. Counselling on stigma and disclosure were also reported to reduce refusal of linkage to care due to nondisclosure and fear of stigma.
	Lifson AR, 2017. Ethiopia (39). Panel/longitudinal.	No client was loss to follow up in the project, with positive client-reported outcomes supporting findings that intervention can improve retention in care.
	Maulsby C, 2015, USA (40). Panel/longitudinal.	Evidence of improvement reported, with 69% of participants retained in care at follow up. Older participants were more likely to be engaged and retained in care. Differences by race and gender in HIV care varied across programs, reflecting the diverse target populations, locations, and strategies employed by different sites.
	Monroe A, 2017, Uganda (41). Qualitative.	Qualitative results demonstrated plausible mechanisms through which peer support improved engagement in care to support findings from pragmatic trial.
	Minick SG, 2018, USA (42). Qualitative.	Peer mentoring was perceived as acceptable and impactful. Intervention was not likely to be intensive or broad enough to overcome stigma, low motivation and structural barriers to improving reengagement and retention in care, which may explain lack of effect reported in RCT.
	Myers JJ, 2018, USA (43). RCT.	Participants were more likely to be consistently engaged in HIV care relative to control group.
	Phiri S, 2017, Malawi (45). RCT.	Retention was higher in facility-based and community-based models compared with standard of care.
	Pitpitan EV, 2020, Mexico (46). Qualitative.	There was consensus that the program could improve ART coverage for key populations by helping to overcome geographic, transportation, and sociostructural barriers to HIV care. Police harassment, mobility, and non-HIV comorbidities were identified as challenges the program would need to navigate.
	Ryerson Espino SL, USA (47). Qualitative.	Out of ten sites all struggled to develop, and only five persisted through challenges to implement peer programs aimed at improving HIV linkage and retention initiative. The paper describes sites’ challenges and facilitators to develop, implement, and evaluate peer roles.
	Reback CJ, USA, 2019 (49). Panel/longitudinal.	Peer navigation combined with incentives was associated with significantly increased behaviours related to linkage and retention in HIV care.
	Reback CJ, 2019, USA (48). Panel/longitudinal.	Peer health navigation sessions were positively related to the number of HIV care visits for users of methamphetamine and any stimulant.
	Sam-Agudu NA, 2017, Nigeria (50). Non-randomised control trial.	Structured peer support significantly improved postpartum retention in care.
	Steward WT, 2018, South Africa (53). Mixed methods.	Program assessed as a feasible and acceptable approach for promoting engagement in care, with qualitative findings demonstrating mechanisms through which peer support assisted participants to overcome barriers to care related to stigma and discrimination, such as HIV disclosure.
Health service engagement	Chevrier C, 2016, India (31). Qualitative.	Findings provided detailed descriptions of how program activities intervened in discrimination, excluding participants from full participation in healthcare settings including ART centres, private and public hospitals.
Virological suppression.	Cabral H, 2018, USA (28). RCT.	No difference in viral load suppression between intervention and standard of care groups. For those who completed all educational sessions in the intervention model there was a suggestive improvement.
	Cunningham W, 2018, USA (32). RCT.	Intervention was successful at preventing declines in viral suppression, typically seen after release from incarceration, compared with standard transitional case management. The intervention was most effective at 12 months among the homeless and those who were virally suppressed at baseline.
	Giordano TP, 2016, USA (33). RCT.	The peer mentoring intervention was no more successful in improving virologic status than the control.
	Griffith D, 2019, USA (35). Pragmatic trial.	Improved retention in care did not to lead to improved virologic suppression.
	Hosseinipour M, 2017 Malawi (36) RCT.	Virological suppression did not differ according to treatment support arm.
	Maulsby C, 2015, USA (39). Panel/longitudinal.	Evidence of improvement reported with 46% of participants virally suppressed at follow-up. Older participants were more likely to be virologically suppressed.
	Minick SG, 2018, USA (42). Qualitative.	Peer mentoring was perceived as acceptable and impactful. Intervention was not likely to be intensive or broad enough to overcome stigma, low motivation and structural barriers to improving reengagement and retention in care, which may explain lack of effect on viral suppression reported in RCT.
	Myers JJ, 2018, USA (43). RCT.	There were no significant differences between groups in achieving undetectable viral load at study end or sustained suppression during the follow-up period.
	Reback CJ, USA, 2019 (49). Panel/longitudinal.	Peer navigation combined with incentives was associated with a significantly increased probability of achieving modest reductions in viral load and reaching and sustaining an undetectable viral load.
	Reback CJ, 2019, USA (48). Panel/longitudinal.	Peer health navigation sessions were positively related to reductions in viral load and reaching and sustaining an undetectable viral load for users of methamphetamine and any stimulant.
	Sam-Agudu NA, 2017, Nigeria (50). Non-randomised trial.	Structured peer support significantly improved rates of undetectable viral loads among women.
ART initiation and adherence	Cataldo F, 2017, Malawi (29). Qualitative.	Identified a need for patient education and psychosocial support with respect to the immediacy of ART initiation on the day of HIV diagnosis and disclosure to husbands and male partners. Participants were generally welcoming of peer support but concerned about confidentiality and stigma.
	Chang LW, 2015, Uganda (30). Randomised pragmatic trial.	No intervention effects were observed on ART initiation.
	Phiri S, 2017, Malawi (31).	ART uptake was higher in facility-based and community-based models compared with standard of care.
	Karwa R, 2017, Kenya (37). Mixed methods.	Providing medication refills for patients unwilling to disclose to medical teams and nurses was a clear need peer navigators met.
	Monroe A, 2017, Uganda (41). Qualitative.	Results identified challenges which explain lack intervention effect on ART initiation, including insufficient messaging surrounding ART initiation, lack of care continuity after ART initiation, rare breaches in confidentiality, and structural challenges.
	Graham SM, 2015, Kenya (34). Qualitative.	Describes the development of an adherence support intervention tailored for Kenyan MSM assessed as well tolerated, feasible, and acceptable in the pilot phase.
	Reback CJ, USA, 2019 (49). Panel/longitudinal.	Peer health navigation combined with incentives was associated with the sustainment of medication adherence to the achievement and maintenance of virological suppression.
	Reback CJ, 2019, USA (48). Panel/longitudinal.	Peer health navigation sessions were positively related to ART adherence to the achievement and maintenance of virological suppression for users of methamphetamine and any stimulant.
	Steward WT, 2018, South Africa (53). Mixed methods.	Program assessed as a feasible and acceptable approach for promoting ART adherence, with qualitative findings demonstrating mechanisms through which peer support assisted participants to overcome barriers to adherence related to stigma and discrimination such as HIV disclosure.
HIV prevention	Chang LW, 2015, Uganda (30). Randomised pragmatic trial.	Participants in the peer support intervention arm were more likely to report use of cotrimoxazole prophylaxis, and adherence to safe water vessel. No intervention effects were observed on bed net use, or condom use and number of sexual partners.
	Monroe A, 2017, Uganda (41). Qualitative.	Qualitative results demonstrated plausible mechanisms through which peer support improved use of cotrimoxazole prophylaxis and adherence to safe water vessel reported in pragmatic trial. Gender and employment reported as structural barriers to changing condom use and number of sexual partners.
	Myers JJ, 2018, USA (43). RCT.	Intervention successful in reducing sex that risks HIV transmission among participants compared with standard of care.
	Odiachi A, 2020, Nigeria (44). Mixed methods.	Attention to expert and mentor mothers’ coping skills and disclosure status, particularly to mentored clients is important to maximize the impact of peer support in prevention of mother to child transmission.
	Sam-Agudu NA, 2017, Nigeria (51). Non-randomised control trial.	Closely supervised, organized mentor mother support significantly improved presentation for early infant diagnosis among HIV-exposed infants in a rural Nigerian setting.
	Steward WT, 2018, South Africa (53). Mixed methods.	Program assessed as a feasible and acceptable approach for promoting HIV prevention, with qualitative findings demonstrating mechanisms through which peer support improved knowledge of condom use and addressed barriers to use of safer sex practices, such as HIV disclosure.
Community and social support engagement	Chevrier C, 2016, India (31). Qualitative.	Findings provided detailed descriptions of how peer-based approach provided a valued source of social support when discrimination excluded participation in families and communities, workplaces, and other HIV support groups and networks.
Cancer prevention	Koneru A, 2017, Tanzania (38). Cross-sectional.	Design and activities of a proposed peer navigation program was highly acceptable approach to address barriers to cervical cancer screening and treatment.
Alcohol and other drug risk behaviours	Myers JJ, 2018, USA (43). RCT.	No statistically significant differences in alcohol and drug use risk behaviour between treatment groups.

As Table 6 shows, the most common health outcomes papers reported on related to linkage to and retention in HIV care, followed by improvement in virological suppression and ART initiation or adherence. This included five papers reporting on results from RCTs, of which one found evidence of greater virological suppression when compared to controls while four did not. Three out of five papers reporting results from RCTs saw greater uptake and retention in care compared to controls, and one out of three reported greater uptake and adherence of ART. All pragmatic trials, quasi-experimental and longitudinal or panel designs found evidence of improvement in continuum of care outcomes, including virological suppression, ART initiation or adherence and retention in care. In one trial improved retention in care did not lead to better virological suppression. In another, stronger retention in care was observed but improved ART initiation was not.

**Table 6:** Secondary health outcomes

Outcome area	Study	Outcome reported
ART initiation and adherence	Cunningham W, 2018, USA (32). RCT.	No improvement in self-reported adherence.
	Giordano TP, 2016, USA (33). RCT.	No significant differences between peer mentoring intervention and control in participants prescribed, taking or adherent to ART.
Health-related quality of life.	Cabral H, 2018, USA (28). RCT.	No significant differences between the peer intervention and standard of care groups.
	Giordano TP, 2016, USA (33). RCT.	No significant differences between peer mentoring intervention and control in health-related quality-of-life measures.
	Lifson AR, 2017. Ethiopia (39). Panel/longitudinal.	Participants had documented improvements in mental health parameters including feelings of internalized stigma and perceived social support. Participants also had a number of positive changes in physical health with increase in CD4 count, BMI, and physical QOL scores and a decrease in symptoms of chronic illness, which likely reflects the benefits of ART and other clinical health interventions.
	Minick SG, 2018, USA (39).	Suggested improvements to the intervention included more frequent contact with interventionists to provide additional support for mental health problems and targeting overall health rather than a more selective focus on HIV.
HIV self-management	Cabral H, 2018, USA (28). RCT.	No significant differences between the peer intervention and standard of care groups in self-efficacy or HIV knowledge.
	Cunningham W, 2018, USA (32). RCT.	Improved self-reported retention and adherence knowledge.
	Lifson AR, 2017. Ethiopia (39). Panel/longitudinal.	Participants had documented increases in HIV treatment knowledge.
Linkage and retention in care	Cunningham W, 2018, USA (32). RCT.	Improved self-reported retention in HIV primary care.
Drug use	Cunningham W, 2018, USA (32). RCT.	There was no effect on reported substance use.
Health service utilisation	Cunningham W, 2018, USA (32). RCT.	Peer navigation arm participants reported a greater increase in mental health, case management, treatment assistance and psychiatric hospital visits and less emergency department visits.
	Giordano TP, 2016, USA (33). RCT.	No significant differences between peer mentoring intervention and control in hopsitalisation or emergency room visits not resulting in admission.
	Myers JJ, 2018, USA (43). RCT.	Those in the intervention arm were also significantly more likely to be linked to mental health and substance dependency treatment.

Of the studies with qualitative components, four papers developed program theory and provided descriptions of the processes and mechanisms that led to effects on HIV care continuum outcomes (see Table 5). Studies also explored the needs of target populations in relation to healthcare engagement and the HIV continuum of care [29, 31, 46] or described enablers and barriers for successful implementation [42, 47, 52]. The feasibility [34, 49], acceptability [29, 34, 46, 49, 53] and safety [34] of programs targeting these outcomes were also assessed, with positive results.

The primary endpoints of six papers also included the prevention of HIV (see Table 5). Of these, one RCT reported a reduction in condomless sex which risked transmission and one pragmatic trial reported no change in condom use or number of partners. Among the two studies that aimed to show the effect of peer navigation programs on behaviours related to HIV prevention during pregnancy, support was shown to promote infant diagnosis [51], and treatment initiation and uptake of maternal prevention programs [45, 50]. Reducing risk behaviours related to drug and alcohol use was a primary outcome of a program investigated by one RCT [43] which found no significant effect when compared to the control group.

#### Secondary health outcomes

Our analysis also identified health outcomes that researchers either considered secondary or viewed in terms of their contribution to HIV care continuum outcomes. Secondary health outcomes are summarised in Table 6. These included health outcomes that would enable greater self-management of HIV, such as improvement in self-efficacy, the uptake of knowledge and skills related to HIV care and treatment and engagement with healthcare professionals and other supports. Of the studies which assessed these outcomes, one panel study found evidence of improvement in HIV knowledge and one out of two papers from RCTs reported improvement when compared to controls, while there was no reported improvement in self-efficacy from the intervention (see Table 6). Two papers based on results from RCTs reported increased engagement in healthcare engagement while one did not. Qualitative and mixed methods studies also described how programs addressed these outcomes.

Table 6 shows that 4 studies detailed how programs addressed or affected factors related to quality of life as secondary outcomes, such as the impact of HIV on general health and function, and the influence of HIV, stigma and discrimination on self-esteem, mental health, social wellbeing and relationships. Of these, two papers utilising RCTs reported no improvement in validated measures of health-related quality of life and one panel study reported improvement in quality of life related to physical health and perceived levels of stigma and social support. One qualitative study developed theory and provided recommendations for the activities and mechanisms that could improve factors related to HIV and quality of life, such as mental health and broader wellbeing (see Table 6).

## Discussion

### Peer navigators and their effects

Our review aimed to clarify peer navigation as a distinct service model for the promotion of health among people living with HIV, offering a synthesis of the form, function and outcomes of programs found in the recently published research literature.

The peer navigation programs captured by our search incorporated key elements of health systems navigation as well as roles traditionally fulfilled by peers to promote the health and wellbeing of people living with HIV. The peer status of navigators was primarily established by living with HIV and navigators often shared sub-group characteristics with target populations. Peer navigators operated in formalised roles, either as employees or volunteers in healthcare settings and community health organisations providing linkage and referral to health services and community and social support. Navigators were selected for the skills and characteristics they were likely to have as peers, rather than formal education or qualifications. Peer navigators were also provided with role-specific training, supervision and support to fulfil their duties. Their activities fell into the broad categories of providing linkage and referrals to health services, liaison and communication between clinical services and clients, practical support and material aid, education and informational support, coaching and skills building, emotional and social support and less frequently, patient advocacy. Within this scope, interventions provided tailored support to individuals and were generally intensive and short-term in nature.

Across the studies in our review, we found strong justification for peer navigators to perform these roles and activities based on explanations of how peer engagement enhanced the effectiveness of programs. The program theories and mechanisms described by researchers centred on the ability of peer navigators to build on their shared experiences, circumstances or affinity with target groups to establish trust, credibility, empathy and understanding or inspire role modelling, motivation and empowerment. These discussions are strongly aligned with the broader literature on the socially supportive role of peers in health promotion [4, 6, 54, 55] and were informed by robust theoretical frameworks including the information-motivation-behavioural skills (IMB) model [56], social support frameworks [57–59] and social learning theory [60], as well as patient-centred, strengths-based and social empowerment perspectives [61, 62], positioning peer navigators to effectively influence a wide-range of desired health behaviours and outcomes.

The studies captured by our review primarily provided evidence of the ability of peer navigation programs to strengthen the HIV continuum of care. Outcomes from more rigorous designs which combined randomisation and control groups were less consistent. Many studies demonstrated how peer navigation programs addressed the prevention of HIV, quality of life, mental health and wellbeing and disease self-management but fewer captured effects or detailed descriptions of the processes through which programs influence these outcomes.

### Priorities for research

The mechanisms and activities of the peer navigation programs we reviewed addressed a wide range of health outcomes and behaviours. While strengthening the continuum of care was often predicated on improvement in areas such as quality of life, mental health and wellbeing, or disease-self management researchers often did not conceptualise these as significant goals in their own right. Unless considered in terms of its contribution to the HIV care continuum, the influence of peer navigation programs on these outcomes were subsequently captured much less consistently. Apart from HIV prevention, substance dependence and screening for cervical cancer, the influence of peer navigation on health promotion and prevention of comorbidities among people living with HIV were not addressed as primary outcomes by the studies captured in this review.

Given the strong theoretical basis set out by researchers for peer navigators to promote health and wellbeing for people living with HIV, there is a significant justification for more research to demonstrate the effects of peer navigation on these health outcomes. Particularly, the detection and treatment of other disease and opportunistic infections, which are among the leading causes of HIV-related mortality globally [63, 64]. Evidence which has only begun to enter the research literature also suggests that chronic healthcare outcomes which reach beyond medical adherence are a priority for peer navigation programs operating in high-income settings which already have strong continuum of care outcomes [65]. With the ultimate goals of successful treatment for any person living with HIV being to prevent death, disease and improve overall health, wellbeing and quality of life, it remains a priority globally to understand how effectively peer navigation can address these outcomes directly.

Our review also demonstrates a need for research to build the evidence base for peer navigation programs operating in diverse settings and healthcare systems. Studies investigated programs proposed for all key populations. Evaluations of programs which targeted all people living with HIV generally provided information about the characteristics and risk-factors of study participants. However, our review shows that this research is concentrated on peer navigation programs operating in large urban centres in the United States, which was the only highly resourced healthcare system investigated by studies in our review. Our search identified research conducted in Mexico, India and seven countries in sub-Saharan Africa. The studies that we reviewed reported that intersecting forms of HIV-based stigma, discrimination, criminalisation, the high cost of healthcare and a lack of an enabling environment for key populations of women, sex workers, gay and bisexual men and men who have sex with men, people who use drugs, ethnic minorities and trans people were barriers to the effectiveness of peer navigation programs and health and wellbeing outcomes for people living with HIV [29, 31, 34, 41, 44, 46, 48, 49, 52]. No other studies targeted regions in which the HIV response has recently been identified as going backwards, such as Eastern Europe, Central Aisa, the Middle East, North Africa and Latin America [8]. Researchers who collaborate with communities in these regions are well positioned to demonstrate how peer navigation programs respond to drivers of the epidemic and HIV-related disparities and inequalities in these systems.

In many places around the world peer-based responses are well-established [1] and initiatives such as peer navigation programs are delivered by peer-led community organisations representing people living with HIV and affected communities [11–16]. However, this, too was not reflected in the research captured in our review. Studies mostly investigated programs operated and managed by clinics employing peer staff. In some cases [27, 35], the scope of programs was limited to direct liaison between clinical providers and their clients, performing tasks such as reminders and follow up for appointments and medical adherence. As a result, the research literature risks constructing a limiting and largely medicalised role for HIV peer navigators in health systems. Peer-led organisations with strong links to local communities and expertise in the delivery of peer-based programs are likely to have different priorities, as well as unique strengths and challenges in implementing peer navigation programs which should be explored [66].

Similarly, the strategies and mechanisms employed by peer navigation programs often imply a multi-sectoral approach. Only five studies, however, investigated programs operated by or in collaboration with community health organisations. Reported collaboration between implementing clinics and community health organisations in our sample was largely limited to the development of referral pathways between healthcare and other social services and the recruitment of peer staff. Peer-based programs are known to drive improvement and enhance the effectiveness of healthcare systems while significant adaptation and engagement between clinical and community partners is likely to be required to deliver programs across clinical and community settings [66]. As community health organisations and third sector NGOs play a large role in the commission and delivery of HIV programs globally, understanding how peer navigation programs respond to and operate in these settings remains a priority.

### Improving evaluation, quality and impact of peer navigation programs

Our review also provides guidance on how researchers can continue to contribute to improving the quality and impact of peer navigation programs.

The strong theorical basis set out by authors to justify peer engagement to deliver health systems navigation represents an advancement in the conceptualisation and design of peer navigation programs and similar peer interventions as described in the research literature. In line with guidance from Simoni et al., [6] researchers should clearly define who peers navigators are, and with reference to an appropriate underlying theoretical framework, provide an explanation of how their characteristics, skills and experiences enhance program activities and desired effects. Researchers who continue to think carefully and conceptually about intervention design will be able to contribute to the development and improvement of approaches for peer navigation to promote a wider range of health outcomes for people living with HIV.

Our recommendations draw attention to how the desired effects of peer navigation programs are identified, measured and evaluated. Our analysis of the activities and mechanisms described in papers affirms that peer navigation programs are inherently complex, social interventions. Programs mediate the intersection between HIV and the psychological, social and healthcare contexts of people living with HIV. Only a small number of studies in our review aimed to capture program effects on culturally and contextually informed assessments of health and wellbeing, such as quality of life. Various measures, including general health-related quality of life scales and items addressing feelings of internalised stigma and perceived social support were used [28, 33, 39]. Use of scales encompassing factors related to HIV, stigma and quality of life may be more sensitive to program effects and assist with collecting consistent data [67]. Qualitative work which captures rich, thick descriptions of how programs operate to influence factors related to quality of life will contribute to valuable theorical development and empirical evidence in this area. So, too, would evaluations that consider systems level effects and types of evidence which more accurately capture the full impact of peer-based approaches, such as the influence of programs on perceived social support, health service and community engagement or HIV-related discrimination, inequities and disparities [66].

As complex social interventions we recommend that future work on peer navigation programs evaluate detailed information about peer navigators, their activities, and the quality of peer engagement. Although peer navigation programs and similar interventions for people living with HIV should be adapted and may differ significantly in their design and conceptualisation our review recommends that, at a minimum, descriptions and proposed mechanisms should incorporate the HIV status of peer navigators and any sub-characteristics or community affiliations relevant to establishing peer status with the target group. The small number of studies which did not include this information were not able provide strong justification for peer engagement or explain its relationship to program activities and effects.

Further, incorporating assessments of how different qualities of peer engagement contribute to or enhance program effects into program evaluation will also support the improvement and implementation of peer navigation programs. For example, in our study most researchers provided at least some information about the mode and duration of peer engagement, but it was only the most detailed experimental designs which considered the influence of the amount of contact on program effects. Studies which evaluated these qualities of peer engagement against study outcomes affirmed that intensive support over a relatively short period of time was required to meet initial support needs, with a more enduring need for social and emotional support and postpartum care [28, 29, 42, 43, 68]. This aligns with findings from the most recent systematic review of peer navigation programs and similar interventions, which found consistent evidence that programs providing intensive, in-person support were effective at improving HIV continuum of care outcomes [7].

Similarly, future work exploring what workplace support structures and community and clinical engagement is required for peer navigators to work most effectively would provide valuable contributions to the evidence base for successful implementation. Most studies in our review reported information about the training, support, supervision, pay and conditions available for peer navigation roles but were less frequently set up to evaluate the quality or influence of these factors on the effectiveness of programs. A common finding among studies which did consider the influence of pay and conditions was that workplaces and employment structures which provided the most stability and flexibility for navigators to meet their own health and wellbeing needs contributed to the successful delivery of programs [32, 43, 47, 52, 68]. There was also evidence that more intensive and structured supervision for peer navigators providing a range of support led to better program outcomes [47, 50].

The majority of programs investigated by studies in our review were managed or delivered in clinical healthcare settings. Challenges noted in these environments included power imbalances and a lack of organisational safety and employment frameworks for navigators to be openly recruited, identify and operate in their capacity as peers [47, 52]. Policy development and training for clinical providers to better understand the values and practices underpinning peer work was recommended by these studies as well as practice-based guidelines and standards, which further emphasise the promotion of GIPA/MIPA principals [11–16]. Notably, our review found limited evidence to inform knowledge of the strengths and challenges of program implementation led by community health organisations and how programs can be delivered in collaboration or adapted to different healthcare settings, organisations and communities. Practice-based guidelines emphasise conducting local needs assessments for both collaboration and adaptation and the importance of organisations to have strong links back to local communities of people living with HIV [11–16]. Examples of the mentorship, training and supervision and support structures that community health organisations can provide are also identified. Future research which further develops this emerging evidence base will significantly inform discussions of the scalability and implementation of peer navigation programs in diverse health systems and HIV responses.

### Limitations

Our review only considered peer navigation programs operating in the current treatment cascade environment since 2015 when global targets were established. Studies conducted previously to this, particularly in the pre-ART era, are likely to consider other health outcomes. English language and the databases we consulted are also likely to have skewed our search towards studies conducted in English speaking countries, particularly the United States. We used broad search terms to capture programs similar in form and function to peer navigation services. Although peer navigation is an increasingly common way in which peer interventions for people living with HIV are conceptualised, our search may have missed programs which draw more strongly from other traditions of peer-based support and mutual aid. Similarly, our review focused on peer navigation interventions providing structured, tailored and in-person support to individuals, however, we recognise that peer support for people living with HIV can exist within many relationships and networks, and provide many benefits to individuals and communities. As we did not assess the risk of study bias, we were unable to report on the reliability of outcomes reported by studies.

## Conclusions

HIV peer navigation incorporates key elements of health systems navigation as well as roles traditionally fulfilled by peers to promote the health and wellbeing of people living with HIV. Recent research provides a strong theoretical basis for peer engagement to enhance the effectiveness of health systems navigation and social support as well as evidence for the ability of peer navigation to strengthen the HIV continuum of care. However, the scope of inquiry remains limited. More research is required to capture the full impact and role that peer navigation programs may play in the detection and prevention of opportunistic infections and health promotion for non-communicable disease and quality of life concerns for people living with HIV in diverse settings, populations, implementing organisations and healthcare systems. Peer navigation programs are complex, social interventions. We recommend that future work continue to evaluate detailed information about HIV peer navigators, their activities, the quality of peer engagement as well as employment and community support structures to improve quality and impact.

## Electronic supplementary material

Below is the link to the electronic supplementary material.

Supplementary Material 1

## Data Availability

Not applicable.

## References

[1] Brown G, O’Donnell D, Crooks L, Lake R (2014). Mobilisation, politics, investment and constant adaptation: lessons from the Australian health-promotion response to HIV. Health Promot J Aust Off J Aust Assoc Health Promot Prof.

[2] UNAIDS. Policy Brief The Greater Involvement of People Living with HIV (GIPA) [Internet]. Geneva: UNAIDS; 2007 Apr [cited 2020 May 25] p. 4. Available from: https://www.unaids.org/en/resources/documents/2007/20070410_jc1299-policybrief-gipa_en.pdf.

[3] Simoni JM, Nelson KM, Franks JC, Yard SS, Lehavot K (2011). Are peer interventions for HIV efficacious? A systematic review. AIDS Behav.

[4] Dennis C-L. Peer support within a health care context: a concept analysis. Int J Nurs Stud. 2003 Mar 1;40(3):321–32. 10.1016/s0020-7489(02)00092-5 12605954

[5] Webel AR, Okonsky J, Trompeta J, Holzemer WL (2010). A Systematic Review of the Effectiveness of Peer-Based Interventions on Health-Related Behaviors in Adults. Am J Public Health.

[6] Simoni JM, Franks JC, Lehavot K, Yard SS (2011). Peer Interventions to Promote Health: Conceptual Considerations. Am J Orthopsychiatry.

[7] Berg RC, Page S, Øgård-Repål A (2021). The effectiveness of peer-support for people living with HIV: A systematic review and meta-analysis. PLoS ONE.

[8] UNAIDS Joint United Nations Programme on HIV/AIDS. 2025 AIDS Targets [Internet]. Geneva: UNAIDS Joint United Nations Programme on HIV/AIDS; 2020 [cited 2021 Nov 23]. Available from: https://aidstargets2025.unaids.org/.

[9] Bradford JB, Coleman S, Cunningham W. HIV System Navigation: An Emerging Model to Improve HIV Care Access. AIDS Patient Care STDs. 2007 Jun 1;21(s1):1–49. 10.1089/apc.2007.9987 17563290

[10] Mizuno Y, Higa DH, Leighton CA, Roland KB, Deluca JB, Koenig LJ (2018). Is HIV patient navigation associated with HIV care continuum outcomes?. AIDS Lond Engl.

[11] Koester KA, Morewitz M, Pearson C, Weeks J, Packard R, Estes M (2014). Patient navigation facilitates medical and social services engagement among HIV-infected individuals leaving jail and returning to the community. AIDS Patient Care STDs.

[12] Farrisi D, Dietz N (2013). Patient navigation is a client-centered approach that helps to engage people in HIV care. HIV Clin.

[13] Broeckaert L. Practice Guidelines in Peer Navigation for People Living with HIV [Internet]. CATIE - Canadian AIDS Treatment Exchange; 2018. Available from: www.catie.ca.

[14] National Association of People Living with HIV Australia (NAPWHA). Australian HIV Peer Support Standards [Internet]. Newtown: National Association of People Living with HIV Australia (NAPWHA); 2020 [cited 2021 Nov 19]. Available from: https://napwha.org.au/resource/australian-hiv-peer-support-standards/.

[15] Positively UK. National Standards for Peer Support in HIV [Internet]. London; 2017 [cited 2021 Nov 19]. Available from: http://hivpeersupport.com/.

[16] NMAC (2015). HIV Navigation Services: A Guide to Peer and Patient Navigation Programs [Internet].

[17] FHI360 (2017). Peer Navigation for Key Populations: Implementation Guide [Internet].

[18] AIDS United (2015). Best Practices for Integrating Peer Navigators into HIV Models of Care. [Internet].

[19] Genberg BL, Shangani S, Sabatino K, Rachlis B, Wachira J, Braitstein P (2016). Improving Engagement in the HIV Care Cascade: A Systematic Review of Interventions Involving People Living with HIV/AIDS as Peers. AIDS Behav.

[20] Boucher LM, Liddy C, Mihan A, Kendall C. Peer-led Self-management Interventions and Adherence to Antiretroviral Therapy Among People Living with HIV: A Systematic Review. AIDS Behav. 2020 Apr 1;24(4):998–1022. 10.1007/s10461-019-02690-7 31598801

[21] UNAIDS Joint United Nations Programme on HIV/AIDS. Fast-Track - Ending the AIDS epidemic by 2030 [Internet]. Geneva: UNAIDS Joint United Nations Programme on HIV/AIDS; 2014 [cited 2021 Nov 23]. Available from: https://www.unaids.org/en/resources/documents/2014/JC2686_WAD2014report.

[22] 90-90-90. treatment target [Internet]. [cited 2022 Mar 21]. Available from: https://www.unaids.org/en/90-90-90.

[23] Lazarus JV, Safreed-Harmon K, Barton SE, Costagliola D, Dedes N, del Amo Valero J (2016). Beyond viral suppression of HIV – the new quality of life frontier. BMC Med.

[24] Levac D, Colquhoun H, O’Brien KK (2010). Scoping studies: advancing the methodology. Implement Sci.

[25] Arksey H, O’Malley L (2005). Scoping studies: towards a methodological framework. Int J Soc Res Methodol.

[26] Tricco AC, Lillie E, Zarin W, O’Brien KK, Colquhoun H, Levac D (2018). PRISMA Extension for Scoping Reviews (PRISMA-ScR): Checklist and Explanation. Ann Intern Med.

[27] Adams JA, Whiteman K, McGraw S (2020). Reducing Missed Appointments for Patients With HIV: An Evidence-Based Approach. J Nurs Care Qual.

[28] Cabral HJ, Davis-Plourde K, Sarango M, Fox J, Palmisano J, Rajabiun S. Peer Support and the HIV Continuum of Care: Results from a Multi-Site Randomized Clinical Trial in Three Urban Clinics in the United States. AIDS Behav. 2018 Aug 1;22(8):2627–39. 10.1007/s10461-017-1999-8 29306990

[29] Cataldo F, Chiwaula L, Nkhata M, van Lettow M, Kasende F, Rosenberg NE (1999). Exploring the Experiences of Women and Health Care Workers in the Context of PMTCT Option B Plus in Malawi. J Acquir Immune Defic Syndr.

[30] Chang LW, Nakigozi G, Billioux VG, Gray RH, Serwadda D, Quinn TC (2015). Effectiveness of peer support on care engagement and preventive care intervention utilization among pre-antiretroviral therapy, HIV-infected adults in Rakai, Uganda: a randomized trial. AIDS Behav.

[31] Chevrier C, Khan S, Reza-Paul S, Lorway R (2016). ‘No one was there to care for us’: Ashodaya Samithi’s community-led care and support for people living with HIV in Mysore, India. Glob Public Health.

[32] Cunningham WE, Weiss RE, Nakazono T, Malek MA, Shoptaw SJ, Ettner SL (2018). Effectiveness of a Peer Navigation Intervention to Sustain Viral Suppression Among HIV-Positive Men and Transgender Women Released From Jail: The LINK LA Randomized Clinical Trial. JAMA Intern Med.

[33] Giordano TP, Cully J, Amico KR, Davila JA, Kallen MA, Hartman C (2016). A Randomized Trial to Test a Peer Mentor Intervention to Improve Outcomes in Persons Hospitalized With HIV Infection. Clin Infect Dis Off Publ Infect Dis Soc Am.

[34] Graham SM, Micheni M, Kombo B, Van Der Elst EM, Mugo PM, Kivaya E (2015). Development and pilot testing of an intervention to promote care engagement and adherence among HIV-positive Kenyan MSM. AIDS Lond Engl.

[35] Griffith D, Snyder J, Dell S, Nolan K, Keruly J, Agwu A (2019). Impact of a Youth-Focused Care Model on Retention and Virologic Suppression Among Young Adults With HIV Cared for in an Adult HIV Clinic. J Acquir Immune Defic Syndr 1999.

[36] Hosseinipour M, Nelson JA, Trapence C, Rutstein SE, Kasende F, Kayoyo V, et al. Viral suppression and HIV drug resistance at 6 months among women in Malawi’s Option B + program: Results from the PURE Malawi Study. J Acquir Immune Defic Syndr 1999. 2017 Jun 1;75(Suppl 2):S149–55. 10.1097/QAI.0000000000001368 PMC5431274 28498184

[37] Karwa R, Maina M, Mercer T, Njuguna B, Wachira J, Ngetich C (2017). Leveraging peer-based support to facilitate HIV care in Kenya. PLoS Med.

[38] Koneru A, Jolly PE, Blakemore S, McCree R, Lisovicz NF, Aris EA (2017). Acceptance of peer navigators to reduce barriers to cervical cancer screening and treatment among women with HIV infection in Tanzania. Int J Gynaecol Obstet Off Organ Int Fed Gynaecol Obstet.

[39] Lifson AR, Workneh S, Hailemichael A, Demisse W, Shenie T, Slater L. Implementation of a Peer HIV Community Support Worker Program in Rural Ethiopia to Promote Retention in Care. J Int Assoc Provid AIDS Care. 2017 Feb 1;16(1):75–80. 10.1177/2325957415614648 26518590

[40] Maulsby C, Positive CI, Team, Charles V, Kinsky S, Riordan M, Jain K (2015). Positive Charge: Filling the Gaps in the U.S. HIV Continuum of Care. AIDS Behav.

[41] Monroe A, Nakigozi G, Ddaaki W, Bazaale JM, Gray RH, Wawer MJ (2017). Qualitative insights into implementation, processes, and outcomes of a randomized trial on peer support and HIV care engagement in Rakai, Uganda. BMC Infect Dis.

[42] Minick SG, May SB, Amico KR, Cully J, Davila JA, Kallen MA (2018). Participants’ perspectives on improving retention in HIV care after hospitalization: A post-study qualitative investigation of the MAPPS study. PLoS ONE.

[43] Myers JJ, Kang Dufour M-S, Koester KA, Morewitz M, Packard R, Monico Klein K (2018). The Effect of Patient Navigation on the Likelihood of Engagement in Clinical Care for HIV-Infected Individuals Leaving Jail. Am J Public Health.

[44] Odiachi A, Sam-Agudu NA, Erekaha S, Isah C, Ramadhani HO, Swomen HE (2020). A mixed-methods assessment of disclosure of HIV status among expert mothers living with HIV in rural Nigeria. PLoS ONE.

[45] Phiri S, Tweya H, van Lettow M, Rosenberg NE, Trapence C, Kapito-Tembo A (2017). Impact of Facility- and Community-Based Peer Support Models on Maternal Uptake and Retention in Malawi’s Option B + HIV Prevention of Mother-to-Child Transmission Program: A 3-Arm Cluster Randomized Controlled Trial (PURE Malawi). JAIDS J Acquir Immune Defic Syndr.

[46] Pitpitan EV, Mittal ML, Smith LR (2020). Perceived Need and Acceptability of a Community-Based Peer Navigator Model to Engage Key Populations in HIV Care in Tijuana, Mexico. J Int Assoc Provid AIDS Care.

[47] Ryerson Espino SL, Precht A, Gonzalez M, Garcia I, Eastwood EA, Henderson T (2015). Implementing Peer-Based HIV Interventions in Linkage and Retention Programs: Successes and Challenges. J HIVAIDS Soc Serv.

[48] Reback CJ, Runger D, Fletcher JB. Drug Use is Associated with Delayed Advancement Along the HIV Care Continuum Among Transgender Women of Color. AIDS Behav. 2019;25 Suppl 1(9712133):107-115. 10.1007/s10461-019-02555-z PMC6904536 31187356

[49] Reback CJ, Kisler KA, Fletcher JB. A Novel Adaptation of Peer Health Navigation and Contingency Management for Advancement Along the HIV Care Continuum Among Transgender Women of Color. AIDS Behav. 2019; 25 Suppl 1 (9712133): 40-51. 10.1007/s10461-019-02554-0 PMC6904539 31187355

[50] Sam-Agudu NA, Ramadhani HO, Isah C, Anaba U, Erekaha S, Fan-Osuala C, et al. The Impact of Structured Mentor Mother Programs on 6-Month Postpartum Retention and Viral Suppression among HIV-Positive Women in Rural Nigeria: A Prospective Paired Cohort Study. J Acquir Immune Defic Syndr 1999. 2017;75 Suppl 2(100892005):S173–81. 10.1097/QAI.0000000000001346 28498187

[51] Sam-Agudu NA, Ramadhani HO, Isah C, Erekaha S, Fan-Osuala C, Anaba U, et al. The Impact of Structured Mentor Mother Programs on Presentation for Early Infant Diagnosis Testing in Rural North-Central Nigeria: A Prospective Paired Cohort Study. J Acquir Immune Defic Syndr 1999. 2017;75 Suppl 2(100892005):S182–9. 10.1097/QAI.0000000000001345 28498188

[52] Sam-Agudu NA, Odiachi A, Bathnna MJ, Ekwueme CN, Nwanne G, Iwu EN (2018). “They do not see us as one of them”: a qualitative exploration of mentor mothers’ working relationships with healthcare workers in rural North-Central Nigeria. Hum Resour Health.

[53] Steward WT, Sumitani J, Moran ME, Ratlhagana M-J, Morris JL, Isidoro L (2018). Engaging HIV-positive clients in care: acceptability and mechanisms of action of a peer navigation program in South Africa. AIDS Care.

[54] Dutcher MV, Phicil SN, Goldenkranz SB, Rajabiun S, Franks J, Loscher BS (2011). “Positive Examples”: a bottom-up approach to identifying best practices in HIV care and treatment based on the experiences of peer educators. AIDS Patient Care STDs.

[55] Peterson JL, Rintamaki LS, Brashers DE, Goldsmith DJ, Neidig JL. The Forms and Functions of Peer Social Support for People Living With HIV. J Assoc Nurses AIDS Care. 2012 Jul 1;23(4):294–305. 10.1016/j.jana.2011.08.014 PMC3303966 22079673

[56] Fisher WA, Fisher JD, Harman J. The Information-Motivation-Behavioral Skills Model: A General Social Psychological Approach to Understanding and Promoting Health Behavior. In: Social Psychological Foundations of Health and Illness [Internet]. John Wiley & Sons, Ltd; 2003 [cited 2021 Nov 25]. p. 82–106. Available from: https://onlinelibrary.wiley.com/doi/abs/10.1002/9780470753552.ch4.

[57] Heaney C, Israel BA. Social Networks and Social Supports. In: Karen Glanz, Barbara K. Rimer, K. Viswanath, editors. Health Behavior and Health Education: Theory, Research, and Practice [Internet]. San Francisco, CA: Jossey-Bass; 2008 [cited 2021 Nov 16]. Available from: http://ez.library.latrobe.edu.au/login?url=https://search.ebscohost.com/login.aspx?direct=true&db=nlebk&AN=238450&site=ehost-live&scope=site.

[58] Cohen S, Syme SL (1985). Social support and health.

[59] Kahn R, Antonucci T. Convoys Over the Life Course: Attachment Roles and Social Support. In: Life Span Development. 1980. p. 253–67.

[60] Bandura A. Social foundations of thought and action: A social cognitive theory. Englewood Cliffs, NJ, US: Prentice-Hall, Inc; 1986. xiii, 617 p. (Social foundations of thought and action: A social cognitive theory).

[61] Parker R, Aggleton P (2003). HIV and AIDS-related stigma and discrimination: a conceptual framework and implications for action. Soc Sci Med.

[62] Saleebey D (2013). The strengths perspective in social work practice.

[63] Montales MT, Chaudhury A, Beebe A, Patil S, Patil N (2015). HIV-Associated TB Syndemic: A Growing Clinical Challenge Worldwide. Front Public Health.

[64] Rajasingham R, Smith RM, Park BJ, Jarvis JN, Govender NP, Chiller TM (2017). Global burden of disease of HIV-associated cryptococcal meningitis: an updated analysis. Lancet Infect Dis.

[65] Khalpey Z, Fitzgerald L, Howard C, Istiko SN, Dean J, Mutch A. Peer navigators’ role in supporting people living with human immunodeficiency virus in Australia: Qualitative exploration of general practitioners’ perspectives. Health Soc Care Community [Internet]. 2021 [cited 2021 Aug 3];n/a(n/a). Available from: http://onlinelibrary.wiley.com/doi/abs/10.1111/hsc.13465. 10.1111/hsc.13465 34101291

[66] Brown G, Reeders D, Cogle A, Madden A, Kim J, O’Donnell D A Systems Thinking Approach to Understanding and Demonstrating the Role of Peer-Led Programs and Leadership in the Response to HIV and Hepatitis C: Findings From the W3 Project. Front Public Health [Internet]. 2018 Aug 31 [cited 2019 Oct 24];6. Available from: https://www.ncbi.nlm.nih.gov/pmc/articles/PMC6127267/. 10.3389/fpubh.2018.00231 PMC6127267 30234083

[67] Brown G, Mikołajczak G, Lyons A, Power J, Drummond F, Cogle A, et al. Development and validation of PozQoL: a scale to assess quality of life of PLHIV. BMC Public Health [Internet]. 2018 Apr 20 [cited 2020 May 22];18. Available from: https://www.ncbi.nlm.nih.gov/pmc/articles/PMC5910603/. 10.1186/s12889-018-5433-6 PMC5910603 29678156

[68] Myers JJ, Koester KA, Kang Dufour M-S, Jordan AO, Cruzado-Quinone J, Riker A (2017). Patient navigators effectively support HIV-infected individuals returning to the community from jail settings. Int J Prison Health.

